# Spatial navigation skills in older adults with and without cognitive impairment

**DOI:** 10.3389/fragi.2025.1587003

**Published:** 2025-07-08

**Authors:** Dorota Kossowska-Kuhn, Michael J. Prevratil, Neil Charness, Walter R. Boot, Sara J. Czaja, Wendy A. Rogers

**Affiliations:** ^1^ Department of Psychology, Florida State University, Tallahassee, FL, United States; ^2^ Division of Geriatrics and Palliative Medicine, Center on Aging and Behavioral Research, Weill Cornell Medicine, New York, NY, United States; ^3^ College of Applied Health Sciences, University of Illinois Urbana-Champaign, Champaign, IL, United States

**Keywords:** spatial navigation, wayfinding, mild cognitive impairment, traumatic brain injury, post-stroke

## Abstract

**Introduction:**

Navigation is a fundamental cognitive ability essential for daily functioning. However, navigation skills decline with age and are further impaired in individuals with cognitive impairment (CI). Understanding these deficits is critical for developing interventions to support affected populations.

**Methods:**

This study compared navigation abilities in older adults with CI (n = 20) to a previously collected community-dwelling sample of older adults (n = 380) using a consistent protocol. Both groups completed objective navigation tasks, subjective navigation assessments, and subjective memory evaluations.

**Results:**

Older adults with CI exhibited significantly lower performance on objective navigation tasks and subjective memory assessments compared to the community sample. Among the three subjective navigation measures, only one demonstrated a significant difference between the groups. Additionally, subjective navigation measures were not reliably predicted by subjective memory or objective navigation performance.

**Discussion:**

These findings highlight a unique and complex relationship between navigation, aging, and cognitive impairment. The results underscore the need for further research to explore the effects of different types of CI on navigation and identify strategies to mitigate these deficits.

**Conclusion:**

This study provides valuable insights into navigation impairments associated with cognitive decline in aging populations, paving the way for targeted interventions to preserve navigation skills in affected individuals.

## 1 Introduction

Spatial navigation is essential for daily functioning and represents a multifaceted psychological construct, encompassing components such as visuospatial memory, spatial orientation, spatial computations, and executive functions (Wolbers and Hegarty, 2010). Spatial navigation forms the foundation for a range of activities that entail moving between different places, including commuting to work, shopping for groceries, and engaging in social events. Consequently, it is closely associated with quality of life, independence, and mobility (van der Ham et al., 2022).

The process of normative aging introduces a range of challenges, both physical (VanSwearingen and Studenski, 2014) and cognitive (Harada et al., 2013; Czaja et al., 2019), that affect navigation skills. In their meta-analysis Techentin and collegues showed that older adults performed worse than younger adults in spatial tests, with a standardized mean difference of d = 1.01 (Techentin et al., 2014). This age-related discrepancy remained consistent across various factors including spatial measure (such as mental rotation, spatial perception, or spatial visualization), timing conditions, and whether the test was administered to a group or an individual, with the more pronounced impact of age on response time.

Age-related changes to spatial ability affect not only general performance outcomes but also impact strategy selection and preferences during spatial tasks. For example, older adults tend to favor egocentric navigation over allocentric strategies (Rodgers et al., 2012; Wiener et al., 2013; Goeke et al., 2015). Egocentric navigation relies on self-centered representations, contrasting with the world-centered representations utilized in allocentric navigation (Colombo et al., 2017). Impairments in allocentric spatial abilities during middle age have been linked to an elevated risk of Alzheimer’s disease (AD) (Ritchie et al., 2018) and are evident in the amnestic version of mild cognitive impairment (MCI; Hort et al., 2007).

Alongside the typical changes in cognition linked with aging, some individuals may have additional cognitive impairment (CI), such as MCI, which denotes a stage wherein cognitive functions fall short of age-related expectations, yet daily functioning remains unimpaired to the extent that the individual does not meet the criteria for a dementia diagnosis (Petersen, 2004). CI symptoms can also arise due to traumatic brain injury (TBI) or after a stroke (post-stroke cognitive impairment; PSCI). Spatial navigation deficits are exacerbated in older adults with MCI (Plácido et al., 2022; da Costa et al., 2020; Tuena et al., 2021), TBI (Skelton et al., 2006; Seton et al., 2023), or PSCI (Ogourtsova et al., 2018).

## 2 Objectives

The current investigation was conducted as a part of the ENHANCE Center (Enhancing Neurocognitive Health, Abilities, Networks, and Community Engagement; NIDILRC #90REGE0012-01-00), a multi-site center focused on older adults with MCI, TBI, and PSCI. The AUGMENT (Augmenting User Geocoordinates and Mobility with Enhanced Tutorials) project investigates how older adults with CI navigate around their environment. During an earlier phase of the AUGMENT project, community-dwelling older adults (n = 380) completed online surveys and assessments targeting subjective and objective facets of navigation ability (Prevratil et al., 2023). At this stage, the primary goals were to validate novel measures and develop a robust protocol for assessing navigation in a sample of CI older adults. The current study is an extension of the protocol utilized by Prevratil and colleagues, aimed at administering an extended version to a population of older adults with CI.

The goal of the current analysis was to investigate the nature of spatial navigation deficits in older adults cognitively impared due to MCI, TBI and PSCI and how those deficits compared to a large sample of community-dwelling older adults. Delving into these questions and comparisons would, in theory, help provide future guidance on the needs and unique difficulties older adults with CI face when navigating. To do so, older adults with CI were recruited and completed the same protocol administered by Prevratil et al. (2023).

## 3 Methods

### 3.1 Participants

A sample of community-dwelling older adults was collected as a part of pilot testing assessment protocols for the first phase of the AUGMENT project (Prevratil et al., 2023). Participants in this sample were recruited from the Institute for Successful Longevity’s (ISL) online registry, a repository of older adults interested in research living in northern Florida. Participants were eligible if they were at least 60 years of age, fluent in English, and had access to the internet. In total, 450 individuals completed the survey. Participant data were removed from analysis due to one or both of the following exclusion criteria: 1) completion time for the spatial orientation test (SOT), Directions and ORienting Assessment (DORA), or overall survey was longer than pre-defined time limit, and 2) participant had missing or incomplete data for the desired analyses. 70 participants were removed from analyses, leaving 380 participants in the community-dwelling sample (for details, see Prevratil et al., 2023). As compensation, participants recruited as a part of this sample were entered into a raffle to receive a $50 gift card.

The CI group participants were recruited from three separate sites: Florida State University (FSU), University of Illinois Urbana-Champaign (Illinois), and Weill Cornell Medicine (WCM). Approval was obtained separately for FSU from the Institutional Review Board (IRB) under number STUDY00001380, for Illinois under the SMART IRB Master Common Reciprocal Institutional Review Board Authorization Agreement, and for WCM from the IRB under number FWA00000093. Participants were recruited through the dissemination of flyers, newspaper and online advertisements, as well as through word of mouth. Materials were provided to healthcare providers for distribution to potential participants who met inclusion criteria. Individuals were eligible if they were 60 years of age or older with cognitive impairment resulting from MCI, TBI, or PSCI. Before being formally recruited, eligible individuals completed the Modified Telephone Interview for Cognitive Status (TICS-M), a widely used screening tool for cognitive decline (Cook et al., 2009). Participants were required to have a TICS-M score between 22 and 37, along with self-reported evidence of a diagnosis of mild cognitive impairment (MCI), traumatic brain injury (TBI), or a history of stroke. In total, 20 participants were recruited across the three sites (see Table 1). All CI participants received $30 for completing the protocol.

**TABLE 1:** Descriptive statistics.

Variable	Cognitively Impaired		Community-Dwelling		Effect Size (*d*)
	*M*	*SD*	*M*	*SD*	
Spatial Orientation Test (SOT)	99.2	26.3	60.4	32.8	1.20*
Directions and Orienting Assessment (DORA)	83.3	17.5	94.4	10.2	1.04*
Subjective Memory Ability	27.6	4.62	32.7	5.09	1.00*
Subjective Severity of Memory Issues	18.3	8.18	11.6	6.12	1.08*
“Feeling Lost” Subscale	10.8	4.48	9.2	3.01	0.51
“Needing Help” Subscale	8.8	3.99	8.3	2.73	0.18
Wayfinding Subscale	30.5	9.17	38.5	6.96	1.12*

Note. Effect sizes represent Cohen’s *d* coefficients. Values with an asterisk (*) indicate significant group differences where *p* < 0.05 (see method for measure details and scoring).

### 3.2 Materials

#### 3.2.1 Pre-interview questionnaires

Pre-Interview Questionnaires were conducted only for the CI sample. The participants were asked questions about the challenges experienced when navigating to familiar and unfamiliar locations, travel restrictions encountered during the COVID-19 pandemic, navigation or transportation problems before the COVID-19 pandemic, the strategies employed for navigation, the use of navigation or transportation aids, and difficulties encountered while using such aids. Next, as a warm-up task, the participants were prompted to think aloud by imagining making a favorite sandwich and describing each step of it. Then, they were presented with a set of scenarios from two popular transportation applications (Google Maps, Uber) and asked to think aloud while performing component tasks for navigation to target locations.

#### 3.2.2 Demographic questionnaires

As a part of the protocol, both groups were asked general questions regarding demographics including age, gender, education, race and ethnicity, and income.

#### 3.2.3 Self-reported memory performance

Participants were instructed to assess both their perceived memory ability and the severity of any current memory issues using items extracted from the “Metamemory Questionnaire” (Gilewski, Zelinski and Schaie, 1990). For self-reported memory performance, six statements were provided, and participants rated their applicability on a 7-point Likert scale ranging from 1 (“Not at all”) to 7 (“Fully”). Possible scores range from 6 to 42, with higher scores indicating better perceived memory ability. Regarding the severity of current memory issues, an additional six statements were presented for participants to rate on a 7-point Likert scale, where 1 represented “Not serious” and 7 represented “Very serious.” Scores could range from 6 to 42, with higher scores signifying more pronounced memory issues.

#### 3.2.4 Spatial orientation test

The Spatial Orientation Test (SOT; Hegarty and Waller, 2004) served as an objective assessment of spatial ability, requiring participants to orient themselves within a two-dimensional space. In this task, participants receive an image depicting various objects in the environment (such as a cat, a car, a flower, etc.). Participants are prompted to imagine they are standing at the location of one object, facing another object, and then must draw the angle at which a third object is positioned relative to their imagined standpoint. The SOT is comprised of twelve questions with a time limit of 10 minutes. For the present project, a digital adaptation of the SOT was utilized (Friedman et al., 2020).

#### 3.2.5 The directions and ORienting assessment

As part of the AUGMENT Project, a new objective measure of spatial orientation was created. This initial version, known as the DORA, instructs participants to follow a set of directions leading to a designated intersection in a hypothetical neighborhood on a screen display. Upon reaching the final destination, participants must choose the building closest to their position from a multiple-choice selection. The DORA is comprised of two subsections, distinguished by instructional variations. The first subsection provides directions using cardinal points (e.g., go north on fifth Street, east on seventh Avenue, etc.), and intends to engage the allocentric representation of space. Conversely, the second subsection employs left and right instructions (e.g., turn left on fifth Street, turn right on seventh Avenue, etc.), aiming to activate the egocentric representation of space. Each of these subsections is comprised of five questions, for a combined total of ten questions.

#### 3.2.6 Navigation ability

To assess participants’ self-perceived navigation skills, three novel subscales were administered. Questions for these subscales were drawn from the “Wayfinding Questionnaire” (de Rooij et al., 2019; van der Ham et al., 2013) and the “Santa Barbara Sense of Direction Questionnaire” (Hegarty et al., 2002).

In the first subscale, participants were queried about how frequently they experienced a sense of being lost while navigating various environments. This subscale comprised six items, with the size of the environment progressively increasing from “Your yard, parking lot, or area surrounding your home” to “Your region.” Participants used a 5-point Likert scale, where 1 represented “Never” and 5 represented “Always.” A higher score on the “Feeling Lost” subscale indicated greater difficulties with navigation.

For the second subscale, participants were asked about the frequency with which they sought assistance from others while navigating similar environments as in the “Feeling Lost” subscale. The scoring method remained consistent. Consequently, a higher score on the “Needing Help” subscale signified a general inclination toward seeking assistance during navigation.

The third subscale, termed the Wayfinding subscale, featured a condensed set of seven items, each inquiring about the applicability of a given statement to the participant (ex., “I am afraid of losing my way in a new location” and “I can easily find the shortest route to a known destination”). Participants rated each item on a 7-point Likert scale, where 1 denoted “Not at all” and 7 denoted “Fully.” Scores ranged from 7 to 49, with a higher score indicating greater confidence in their wayfinding abilities.

### 3.3 Procedure

For the community-dwelling older adults sample, every participant received an email containing an anonymous link to a Qualtrics survey comprising the entire protocol. Participants independently completed the Qualtrics survey without a specified time constraint. Following the provision of electronic informed consent, participants initially filled out the demographic questionnaire section and the self-assessment questionnaires. Subsequently, participants proceeded to complete the SOT, followed by the DORA.

For the CI sample, following the provision of consent, each participant engaged in a Pre-Interview Questionnaire session conducted over Zoom with a trained assessor. The session was recorded. Subsequently, participants took part in a second session, also conducted over Zoom with a trained assessor, during which they completed the demographic questionnaire, self-assessment questionnaires, SOT, and DORA.

## 4 Results

### 4.1 Group comparisons

A series of two-sample t-tests was conducted comparing a healthy sample of community-dwelling older adults (*n* = 380) with a sample of CI older adults (*n* = 20). Table 1 provides groupwise descriptive statistics of the variables analyzed via two-sample t-tests along with effect sizes of comparisons. Using the ‘pwr’ package (Champely et al., 2018), *post hoc* power analyses with a desired power of 0.8 at a significance level of α = 0.05 indicated the ability to detect medium to large effect sizes (*d* = 0.644).

**FIGURE 1:**
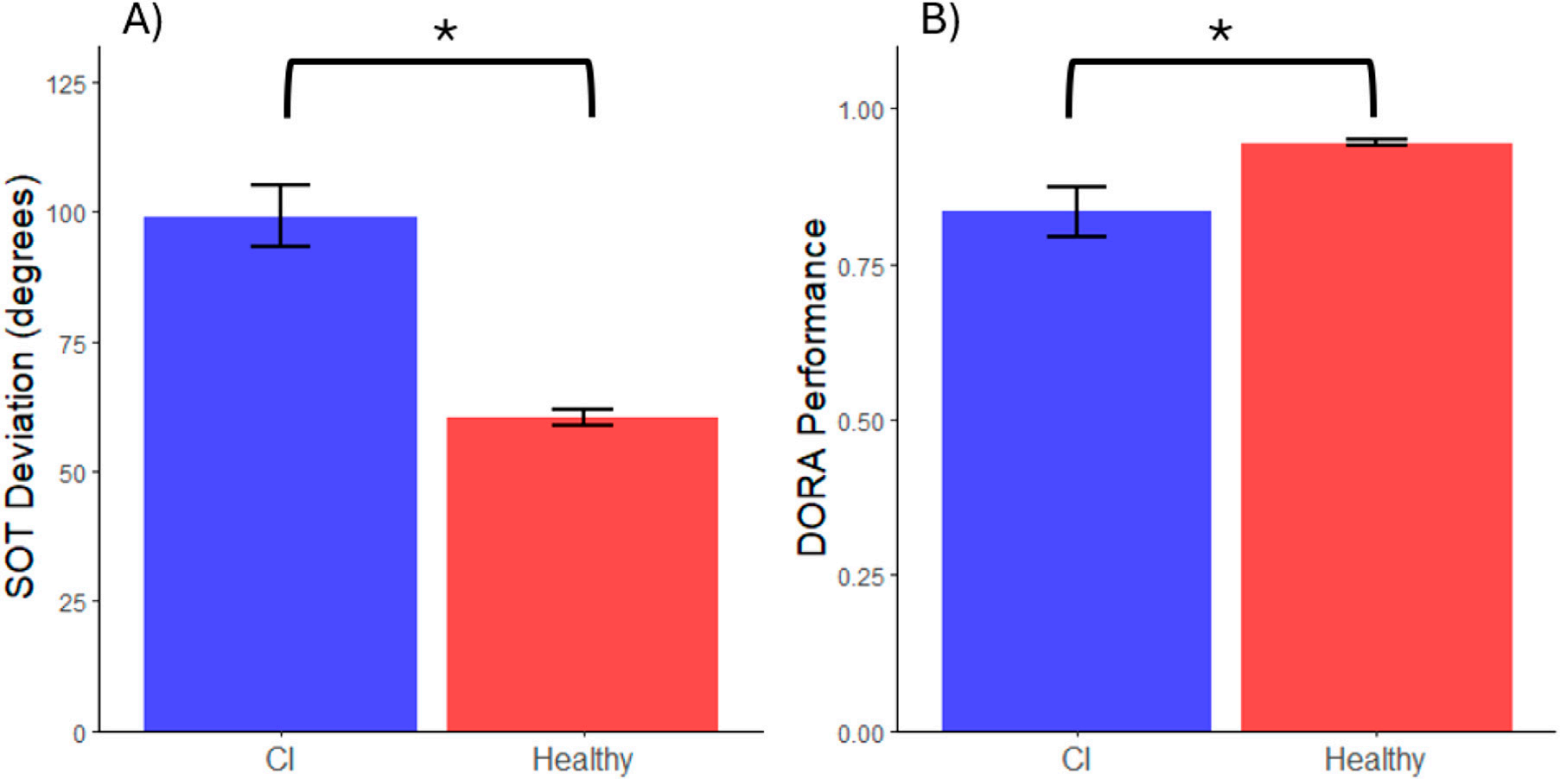
Group Comparisons of Objective Measures of Navigation Ability. Note. * Represents a significant difference between group performance where α = 0.05. Error bars represent one standard error above and below the mean of each group. **(A)** Groupwise average deviation from the correct response on the Spatial Orientation Test, *d* = 1.20. **(B)** Groupwise average percent correct for the Directions and Orienting Assessment, *d* = 1.04.

Referring to the SOT task, the healthy sample had a significantly lower mean angular error than the CI sample which indicates the CI sample pointed less accurately to the target (*t*(20.88) = 6.19, *d* = 1.19, *p* < 0.001; see Figure 1A). On the DORA task, when comparing the healthy sample to the CI sample, the healthy sample had a significantly higher mean than the CI sample, translating to the healthy sample providing more correct answers on the given task (*t*(17.56) = −2.66, *d* = 1.04, *p* = 0.016; see Figure 1B).

**FIGURE 2:**
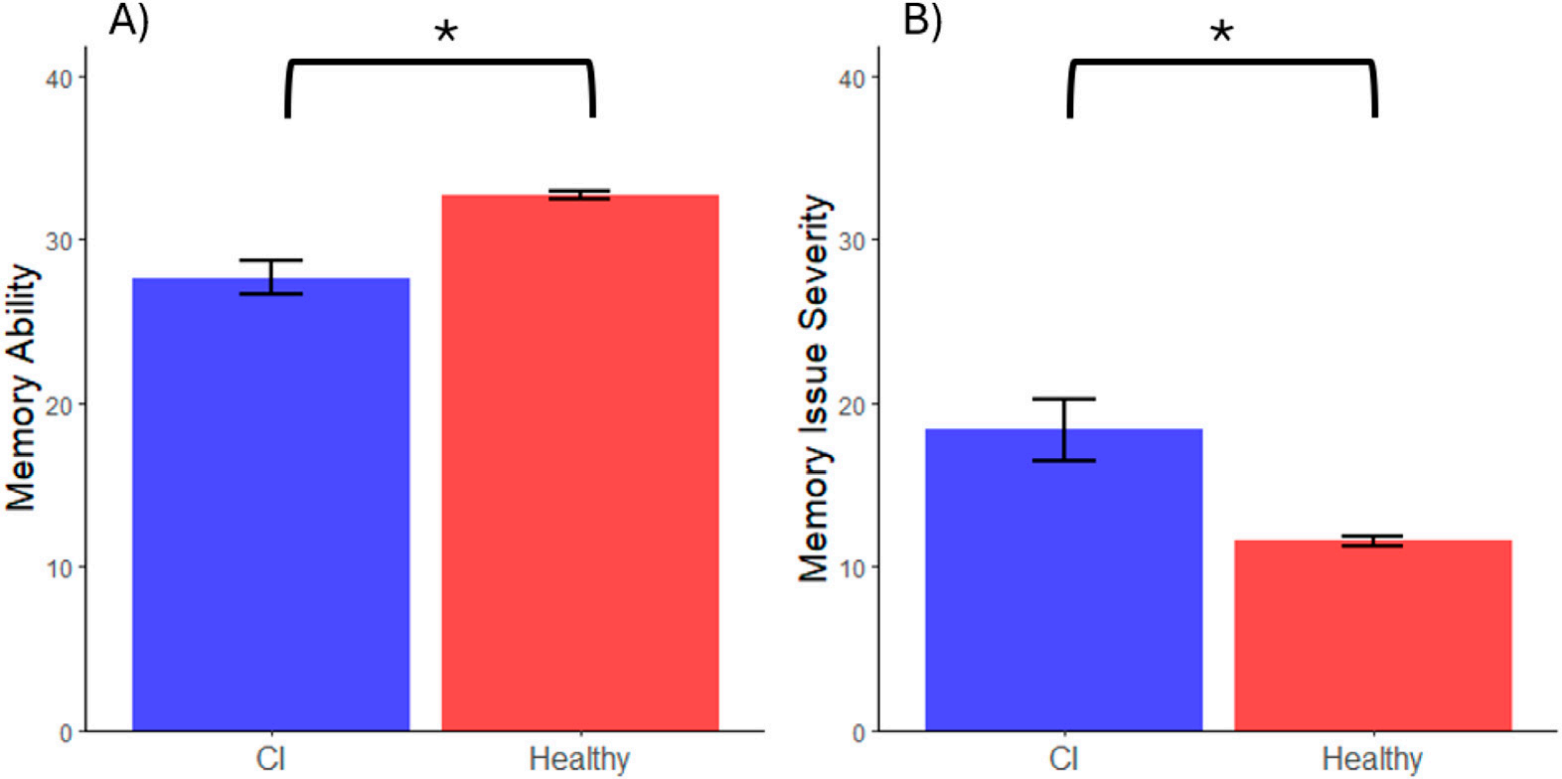
Group Comparisons for Subjective Measures of Memory. Note. * Represents a significant difference between group performance where α = 0.05. Error bars represent one standard error above and below the mean of each group. **(A)** Groupwise average self-rating on memory ability. **(B)** Groupwise average self-rating on severity of memory difficulties.

For subjective memory assessment, the healthy sample reported better outcomes than the CI sample in terms of overall memory ability (*t*(20.24) = −4.64, *d* = 1.00, *p* < 0.001; see Figure 2A). The healthy sample also reported a lower severity of memory difficulties (*t*(17.91) = 3.45, *d* = 1.09, *p* = 0.003; see Figure 2B).

**FIGURE 3:**
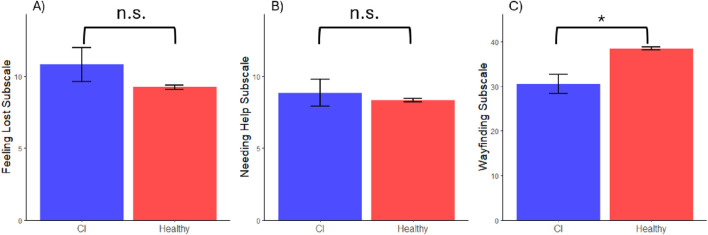
Group Comparisons for Subjective Navigation Ability. Note. * Represents a significant difference between group performance where α = 0.05. n.s. represents a nonsignificant group difference. Error bars represent one standard error above and below the mean of each group. **(A)** Groupwise average self-rating on the “Feeling Lost” subscale. **(B)** Groupwise average self-rating on the “Needing Help” subscale. **(C)** Groupwise average self-rating on the Wayfinding subscale.

When investigating subjective navigation ability, the two-sample t-test showed the healthy sample reported better capabilities on the Wayfinding subscale compared to the CI sample (*t*(19.05) = −3.72, *d* = 1.12, *p* = 0.001; see Figure 3C). There was no significant difference between the healthy sample and the CI sample on the “Needing Help” subscale (*p* = 0.60; see Figure 3A) nor the “Feeling Lost” subscale (*p* = 0.15; see Figure 3B).

### 4.2 Regression analysis

To replicate the process used by Prevratil et al. (2023), three multiple regression analyses were used to predict each of the three navigation subscales within the CI sample. Those predictors were SOT performance, DORA performance, subjective memory ability, and subjective severity of memory issues. Using the ‘pwr’ package (Champely et al., 2018), *post hoc* power analyses indicated that, when including these four predictors and a desired power of 0.8 at a significance level of α = 0.05, regressions were powered enough to detect only the strongest of predictors (*f* ^*2*^ = 0.815).

When predicting general wayfinding in the CI sample, the overall model was not significant using the predictors listed above (R^2^_adj_ = 0.03, *p* = 0.39) and, consequently, none of the predictors were significant (all *p*’s > 0.05). The same general outcome was found when predicting the “Needing Help” subscale (R^2^_adj_ = 0.06, *p* = 0.35). For the “Feeling Lost” subscale, the overall model was significant (R^2^_adj_ = 0.48, *p* = 0.015). Only one predictor was significant: subjective memory ability (*t* = −2.42, *β* = −0.447, *p* = 0.03), indicating that a higher feeling of being lost when navigating is related to lower subjective assessment of one’s memory ability.

## 5 Limitations

One important limitation of this study is the small size of the cognitively impaired (CI) sample (n = 20), which introduces statistical power limitations. While the healthy sample (n = 380) was sufficiently large to detect small effect sizes with accuracy, comparisons between the healthy and CI groups were limited to detecting moderate effects (d = 0.644), and our ability to detect anything but strong associations in the regression analyses was substantially constrained (f^2^ = 0.815). Although most group comparisons were robust and significant, the “Feeling Lost” and “Need Help” subscales did not show significant group differences. A larger CI sample may have allowed us to detect such differences; indeed, the calculated Cohen’s d for the “Feeling Lost” subscale was 0.51 (see Table 1), suggesting a moderate effect that may not have reached significance due to limited power. In addition, the absence of significant group differences on these two subscales may also reflect limitations in the reliability and validity of the self-report measures themselves when used with CI populations. This is a common issue in the field, as many widely used questionnaires have not been formally validated in older adults with cognitive impairment. Further validation work is needed to ensure that such subjective measures accurately capture navigation difficulties in this population. Moreover, with a larger CI sample, it is possible that additional predictors would have emerged as significant in the regression analyses. Future research should prioritize recruiting larger samples of CI older adults to improve power and sensitivity to smaller but meaningful effects. Given that small sample sizes are typical in the literature on navigation and cognitive impairment (e.g., Lesk et al., 2014; Lee et al., 2014; Tippett et al., 2009; Boccia et al., 2016), meta-analytic techniques may provide a valuable approach for identifying subtle effects that are difficult to detect in individual studies.

Another limitation concerns differences in assessment administration across groups. The community-dwelling sample of older adults completed the assessments independently online, while the CI group was assessed via Zoom sessions, due to COVID-related constraints. This variation in testing environments may introduce methodological biases, such as potential effects of the assessment context on task performance. Although we do not believe this substantially impacted the results—since significant group differences were still observed on key measures—this factor should be considered when interpreting the findings and addressed in future studies through standardized administration procedures.

Another important consideration is that different strategies can be employed in human spatial navigation—specifically, object-centered versus self-centered strategies. We fully acknowledge that this is an important perspective in spatial navigation research. However, differentiating between these strategy types was not within the scope of the current study. We note that this as a valuable direction for future research.

## 6 Discussion

With the current investigation, older adults with CI were compared with a sample of community-dwelling older adults previously collected (Prevratil et al., 2023) utilizing variations of the same study protocol. All participants completed two objective measures of navigation ability, three subjective measures of navigation ability, and two subjective measures of memory. As these novel subjective measures demonstrated valid and reliable results (Prevratil et al., 2023), comparison with a cognitively impaired sample was deemed suitable. The current findings show a nuanced relationship between CI and community sample. While the CI sample reported worse general wayfinding ability and performed poorer on objective navigation metrics, there was no group difference based on one’s need for help or feeling of being lost when navigating.

With the regression analyses, only subjective memory was significantly predictive of one’s feeling of being lost while navigating. No other predictors were significant in any of the three regressions. There are several possible explanations for these results. It is possible that more of these predictors are significant, but the current study was too underpowered to detect these smaller effects. Using the findings of Prevratil et al. (2023) as an example comparison, SOT was a significant predictor in each of the three original regressions but was always the weakest (e.g., it had the lowest reported *β* values). Another possibility is that, for older adults with CI, factors that affect navigation shift significantly.

When comparing outcomes on the subjective assessments between the CI and community groups, the findings imply a partial decline in metacognitive abilities among individuals with CI, where metacognition refers to the capacity to evaluate diverse cognitive skills. For navigation, objective measures (e.g., SOT and DORA) were both worse in the CI sample whereas only one of the subjective measures (e.g., the Wayfinding Subscale) showed a significant difference. This observation aligns with a segment of the current literature on metacognition in CI, as supported by Fitzgerald et al. (2022) and Ownsworth (2015) in the context of TBI. However, it deviates from the perspective presented by Seelye et al. (2010) and Pennington et al. (2021) concerning MCI. These variations in findings may arise due to differences in the definition of metacognition and the array of assessment tools employed, as concluded by Al Banna et al. (2015) in their literature review on metacognition in post-stroke patients.

## 7 Conclusion

Our study contributes valuable insights into how CI may affect navigation ability, but several limitations should be acknowledged. With the relatively small sample size of the CI group, it is not feasible to tease out any likely differences based on the type of CI one was classified as having. With such a small sample size, inferential statistics are underpowered for detecting other than strong associations. Additionally, these data were collected at a single timepoint, while aging effects and those specific to CI are likely to progress over time. Future research with more robust sample sizes and longitudinal designs could provide a more comprehensive understanding of the dynamic relationship between CI, navigation abilities, and memory over time.

Despite the limitations, comparing adults with CI to community sample is crucial for the next phase of the AUGMENT Project, aimed at designing instructional packages to train the use of navigation software (Google Maps, Uber). This comparison identified specific challenges faced by those with CI and established a baseline understanding of their navigation abilities relative to community-dwelling counterparts. Olders adults with CI have expectedly worse performance on navigation abilities, but these detriments may not be fully recognized, as the lack of significant difference on two of the subjective navigation questionnaires demonstrates. The use of objective and subjective measures provided a more complete understanding of the factors influencing navigation performance while highlighting potential relationships between navigation, aging, and cognitive impairment. This finding underscores the need for tailored interventions and tools. Technological interventions could prove highly beneficial given the development of suitable training protocols that take into account specific challenges that people with CI may encounter (ex., memory difficulties). Several training paradigms have been developed and found promising results (Fricke et al., 2022), but the transferability of said interventions to daily life is the subject of continuing discussion.

## Data Availability

The raw data supporting the conclusions of this article will be made available by the authors, without undue reservation.
